# Microbial Contamination and Occurrence of Aflatoxins in Processed Baobab Products in Kenya

**DOI:** 10.1155/2022/2577222

**Published:** 2022-02-25

**Authors:** Margaret James, Willis Owino, Samuel Imathiu

**Affiliations:** Department Food Science and Technology, Jomo Kenyatta University of Agriculture and Technology, P.O. Box 62000-00200, Nairobi, Kenya

## Abstract

Baobab fruit demand has been on the rise in the recent past, and in an attempt to match the demand, farmers and middlemen are forced to harvest immature fruits which are not fully dried. To ensure an acceptable moisture content, baobab fruits are subjected to solar drying, which is a slow process and often carried out in open and unhygienic conditions raising safety concerns. This study was conducted to investigate the microbial and aflatoxin contamination levels in ready-to-eat baobab products from selected formal and informal processors in specific counties of Kenya. Selected processed baobab products were sampled randomly from formal and informal processors and analyzed for the total aerobic count, Enterobacteriaceae, yeast and molds, ergosterol, aflatoxins, moisture, and water activity. The moisture and water activity of baobab pulp and candies from formal processors ranged between 7.73% and 15.06% and 0.532 and 0.740 compared to those from informal processors which ranged from 10.50% to 23.47% and 0.532 to 0.751, respectively. In this study, baobab pulp from formal processors had significantly (*p* = 0.0008, 0.0006) lower Enterobacteriaceae and yeast and molds loads (0.7 ± 0.29 and 3.1 ± 0.38 log 10 CFU/g, respectively) than pulp from informal processors (3.1 ± 0.70 and 5.3 ± 0.11 log 10 CFU/g, respectively). Similarly, the Enterobacteriaceae counts of candies from formal processors (nondetectable) were considerably lower (*p* = 0.015) than those from informal processors (1.8 ± 0.56 log 10 CFU/g). The ergosterol content in these baobab product samples ranged between 0.46 and 1.92 mg/100 g while the aflatoxin content ranged between 3.93 and 11.09 × 103 *μ*g/kg, respectively. Fungal and aflatoxin contamination was detected in 25% and 5% of pulp from formal and informal processors, respectively, and in 5% of candies from informal processors. Microbial contamination in processed baobab products shows an unhygienic processing environment while the fungal and aflatoxin contamination may indicate poor postharvest handling, transport, and storage conditions of baobab fruits along the baobab value chain.

## 1. Introduction

Baobab (*Adansonia digitata*) candies and pulp are ready-to-eat snacks processed from baobab fruit mostly consumed in a number of sub-Saharan Africa countries. The baobab fruit pulp is naturally dried and is rich in vitamin C, calcium, and antioxidants [1]. The presence of baobab fruit pulp increased bioaccessibility of iron from cereals as shown in vitro studies, which may be attributed to high contents of vitamin C and other organic acids [2]. Intake of baobab pulp can has been demonstrated to improve nonheme iron absorption in the populations most vulnerable to iron deficiency [2]. The pulp has prebiotics and inflammation-reduction functions and hence categorized as a functional food [3]. The European Commission and the US Food and Drug Administration classified baobab as a novel food and functional food in the year 2008 contributing to an increase in baobab import volumes due to a significant increase in demand [4]. The rise in baobabs imports led to an increase in demand from African countries with high baobab tree populations, including Kenya, Sudan, Malawi, and Ghana [4, 5].

Baobab tree populations are found abundantly traversing the Kenyan counties of Tharaka Nithi, Kitui, Makueni, Kilifi, Lamu, Kwale, and Taita Taveta. These are semiarid lands which are hotspots for food and nutritional insecurity, as well as high poverty levels. Their livelihood is primarily dependent on subsistence farming, but due to inadequate and unreliable rainfall, harvests from farming activities are quite poor, and relief food is a common feature [6]. In Kenya, baobab is considered a high-priority food tree for future domestication due to its multiple uses [1]. In addition, the baobab value chain has a high potential for product development, value addition, and economic development for the people who inhabit the areas where baobab exists [5]. So far, baobab is only harvested from wild trees, and domestication of the species may increase the quantity and quality of baobab fruit pulp for domestic and export markets [4]. Baobab farmers in the fore-mentioned countries are engaged in trading in baobab pulp albeit with challenges along the baobab value chain. These include poor harvesting practices and drying, as well as poor postharvest handling leading to microbial contamination and overall quality loss.

The baobab fruit dries naturally with maturity, falling off the tree when completely mature and fully dried with a moisture content of about 11% (Chadare et al. [7]). However, market demand has been on the rise in the recent past, and in an attempt to match the demand, farmers and middlemen have resorted to premature harvesting before the fruits are fully dried [8]. Once the baobab pulp has been extracted from fruit, it is generally subjected to solar drying to ensure an acceptable moisture content. Solar drying is a slow process and is often carried out in open and unhygienic conditions [9]. Some of the baobab products derived from baobab pulp are mainly consumed in raw form, without being subjected to any thermal treatments. The final moisture content and hygienic handling conditions during processing determine the safety and degree of deterioration of these products [10].

Some of the handling practices of baobab pulp may also expose the pulp to extrinsic environmental factors such as high humidity. The pulp is hygroscopic and is thus susceptible to moisture absorption leading to mold growth and subsequent spoilage and/or contamination by mycotoxins rendering the commodity unsafe for consumption [10]. Determination of fungal contamination in food products is vital for the safety of the products, and different elements have been suggested as markers for fungal contamination. Ergosterol is a sterol that resides on the cell membranes of fungi and acts in maintaining cell membrane integrity, similar to mammalian cholesterol [11]. Ergosterol qualifies as a marker of fungal contamination in fresh and processed baobab products since it is a constituent of the fungal membrane. It is also environmentally stable and has high reproducibility and sensitivity, thus making it most suitable in comparison with other biomarkers [12].

*Aspergillus parasiticus* and *Aspergillus. flavus* L. strains were recovered from baobab fruit obtained from the market in Zambia, though the average aflatoxin concentrations in the baobab were below maximum allowable levels in food [13]. However, from the study, it was not clear whether the baobab fruits were cracked in the market or in the laboratory to obtain the pulp. There is a high likelihood of baobab contamination along the Kenya baobab value chain considering the current postharvest handling practices. However, there is limited knowledge on the safety of the widely available ready-to-eat processed baobab products. So far, the studies that have been carried out on baobab focus more on the nutritional aspect, utilization as well as economics, market, and trade. This study is aimed at determining the microbial contamination and occurrence of Aflatoxins in selected processed baobab products in three Kenyan counties of Nairobi, Mombasa, and Kilifi.

## 2. Materials and Methods

### 2.1. Sample Size Determination and Sample Collection

This study involved the use of two classification groups: the formal and the informal processors. The term formal was used in this study to indicate processors registered with the Kenya Bureau of Standards (KEBS), whereas informal represents processors not registered with KEBS. Total population sampling was applied for formal processors (control) due to the manageable size of the population and systematic sampling for the informal processors. Details of the formal baobab product processors were obtained from KEBS where a sampling list of 15 processors was obtained with only 10 of the processors selected for this study being fully operational. For formal processors, eight processors were located in Nairobi, one in Kilifi, and one in Mombasa counties. The details of the informal processors were gathered from a baobab processor survey carried out under the project “A value chain analysis of baobab (*Adansonia digitata* L.) products in eastern and coastal Kenya” [14]. The [15] proportion formula below was used to systematically sample a similar number of informal baobab product processors in different counties, whereby two processors were located in Nairobi, three in Mombasa, and five in Kilifi counties.

(1) $$\begin{array}{c} p^{\prime }=\frac{x}{n}, \end{array}$$

*p*′ = proportion ratio, *x* = the size of the proportion, *n* = the size of the total population.

Samples were collected in June and July 2019. Baobab candies and pulp are the highly consumed products and were therefore selected for this study. From each processor, three samples were collected resulting in 60 products. Each sample weighed between 100 and 200 grams. Samples were packed in sterile plastic zip lock bags and appropriately labelled. The bags were placed in cooler boxes with ice packs and transported to the Food Microbiology Laboratory at Jomo Kenyatta University of Agriculture and Technology, for analysis. Samples were stored at 4°C and analyzed within 24 hours.

### 2.2. Determination of Moisture and Water Activity of Baobab Products

All samples were subjected to water activity (*a*_w_) and moisture content measurements. The water activity was measured as described in, using HygroPalm HP23-AW-A Portable Water Activity Analyzer (Rotronic AG, Bassersdorf, Switzerland). The water activity meter was set at *a*_w_Quick mode, and sealed samples were kept at relatively constant temperature (22 ± 3°C) for 5 minutes to allow for the temperature conditions of the sample and the probe to stabilize before the displayed *a*_w_ reading was recorded.

The moisture content was determined as per the AOAC method number IS 4333 [16]. Five grams of baobab samples in triplicates was weighed and placed in a clean dry moisture dish, and the weight of the sample and dish was taken. These were placed in an oven set at a temperature of 105°C and dried for three hours, removed, cooled in a desiccator, and weighed. The amount of moisture in the samples was calculated using the formula:

(2) $$\begin{array}{c} \%\text{Moisture content}=\frac{w1-w2}{w1}\times 100, \end{array}$$

*W*1 = weight before drying, *W*2 = weight after drying.

### 2.3. Microbial Analysis of Baobab Products

The baobab samples were each analyzed in duplicates for the total aerobic count, Enterobacteriaceae, and yeast and molds according to the AOAC microbiological method number ISO 4833, ISO 21528-2, and ISO 7954, respectively [16]. All media was purchased from Sigma-Aldrich (England, UK). Ten grams of the sample was transferred into 90 ml of 0.1% sterile peptone water and mixed uniformly using a bench vortex Mixer® (AHN, Nordhausen, Germany) after which three tenfold serial dilutions (10^−1^ to 10^−3^) were made and analyzed for [17]: total aerobic count, Enterobacteriaceae, and yeast and molds. Total aerobic counts were enumerated on plate count agar (PCA), yeasts and molds on potato dextrose agar (PDA), and Enterobacteriaceae on violet red bile green agar (VRBGA). The spread plate method was used as the plating technique for the three microbial analyses where 0.1 ml of sample serial dilutions was plated. The total aerobic count, Enterobacteriaceae, and yeast and molds counts were assessed after incubating PCA plates at 37°C for 48 hours, VRBGA plates at 37°C for 24 hours, and PDA plates at 25°C for 5 days [17, 18]. The results were expressed as the number of colony-forming units per gram of baobab sample (CFU/g). Data were transformed into logarithm for statistical analysis.

### 2.4. Standard Solutions and Generation of Standard Curves

Ergosterol standard (E6510-5G) was obtained from Sigma-Aldrich (England, UK). The ergosterol stock solution was prepared at a concentration of 1 mg/ml, and further working standards were prepared by serial dilutions with ethanol. A standard calibration curve was then generated, and a correlation coefficient of (*r*^2^) of 0.999 was used to demonstrate linearity. Based on the signal-to-noise ratios (S/N) of 3 : 1 and 10 : 1, respectively, the limit of detection (LOD) and limit of quantification (LOQ) were determined [12]. Precision was calculated as relative standard deviation (%RSD) for repetitive measurements. The aflatoxin standard solution (Fujifilm Wako pure chemical corporation, Osaka, Japan) comprised of aflatoxin mixture of AFB1, AFB2, AFG1, and AFG2 each 25*μ*g/ml in acetonitrile solution. Working standards were prepared by serial diluting with acetonitrile, and a 6-point calibration curve was generated covering the range of 1.25–12.5 ppm for each aflatoxin group.

### 2.5. Ergosterol Analysis of Baobab Products

Extraction and quantification of ergosterol were carried out on each of the 60 baobab samples. Two grams of the sample was placed in the saponification solution composed of 15 ml of methanol and 5 ml of potassium hydroxide solution (40 g/l KOH in ethanol) and agitated for 30 minutes. This was then filtered into evaporation tubes using a 0.2 *μ*m pore size syringe filter. The mixture was evaporated to 1 ml using a gentle stream of nitrogen [11]. The ergosterol concentration was determined by injecting 20 *μ*l of the extract into the HPLC System (Shimadzu Corp., Model LC-20AD/T LPGE, Kyoto, Japan). The HPLC system consisted of an auto-sampler model SIL-20A HT, quaternary pumps (Shimadzu model LC-20 AD), a reverse-phase SUPELCO C-18 (5 *μ*m 260 mm × 4.6 mm) column, CTO-10AVP column oven, and a photodiode detector (SPD-M20A). The separation was achieved through isocratic elution at a flow rate of 2 ml/min, with a linear gradient solvent (methanol : water, 80 : 20, *v*/*v*).

### 2.6. Aflatoxin Analysis of Baobab Products

Two grams of the baobab samples was added to an extraction solution comprising of 1 g sodium chloride in 25 ml (methanol : water 80 : 20 *v*/*v*) and centrifuged at 10,000 rpm for 5 minutes. Five millilitres of supernatant was transferred to a 50 ml centrifuge tube and diluted with 40 ml of 2% PBST (phosphate-buffered saline with tween) solution [19]. The solution was filtered through 0.2 *μ*m millipore filters into vials. Aflatoxin quantification was done by injecting 20 *μ*l of the samples into a SHIMADZU HPLC system (Shimadzu Corp, Model LC-20AD/T LPGE, Kyoto, Japan) equipped with; RF20A fluorescence detector operated at an excitation wavelength of 350 nm and an emission wavelength of 450 nm; auto-sampler SIL 20AHT, a reverse-phase SUPELCO C-18 (5 *μ*m 260 mm × 4.6 mm) column, CTO 10ASVP column oven set at 40°C, and LC20AD quaternary pump. A mobile phase of acetonitrile : methanol : water (10 : 30 : 60 *v*/*v*) was used at a flow rate of 1 ml/min.

### 2.7. Statistical Analysis

Data were analyzed using STATA for windows version 12.1, 2011 package by StataCorp Inc., USA. The data collected from the study were subjected to independent student *T*-test to assess significant differences in water activity, moisture content, and microbial counts between formal and informal processor's products. The microbial data from different regions were also subjected to analysis of variance. Differences among means were separated using the Bonferroni test, and significances were accepted at *p* ≤ 0.05. Spearman's correlation was done to test the correlation between water activity, ergosterol content, and aflatoxin content.

## 3. Results and Discussion

### 3.1. Moisture Content, Water Activity, and Microbial Loads of Baobab Products

Moisture, water activity, and microbial content of baobab pulp and candies from formal and informal processors are shown in (Table 1). The highest moisture content in baobab pulp was 15.60% while that of candies was 23.47%. Baobab candies had relatively high moisture content and water activity as compared to the pulp. During processing of baobab pulp, there is generally no addition of additives or liquids, and the moisture content is dependent on drying processes, transport, and storage conditions [20]. On the other hand, in processing of baobab candies, water is added as a solvent to dissolve additives such as food color and sugar which in turn increases the moisture content (ICRAF [21]). No significant differences were observed in both moisture content and water activity levels between baobab pulps from formal and informal processors. However, the moisture content and water activity of baobab candies from informal processors (17.18 ± 3.8%, 0.704 ± 0.06) were significantly higher (*p* = 0.015, 0.049) than those from formal processors (11.84 ± 2.3%, 0.619 ± 0.10) (Table 1). This could be attributed to poor packaging and storage conditions that expose the candies to high humidity leading to moisture reabsorption. It could also be due to a lack of knowledge among informal baobab processors regarding the existing drying protocol and standards of processing [22]. Water activity is an indicator of free water available in food that supports chemical and biological reactions (United Nations, 2014). The water activity in candies (0.704 ± 0.06) from informal processors was above 0.6 which still provides a suitable environment for the growth of bacteria, yeast, and molds [23].

The presence of microbes in food does not imply unfitness for consumption, but rather an indication of the hygienic status during preparation and processing [24]. Nonetheless, exceeding certain levels such as total aerobic count above 10000 CFU-g, Enterobacteriaceae 100 CFU-g, yeast and molds 100 CFU-g [25], AFB1 2 ppb, and total aflatoxin 4 ppb as permitted for dried fruits can suggest severe cases of poor hygiene and make the food product unfit for consumption [26]. Surpassing the set limits shows a failure to comply with good hygiene practice (GHP) and good manufacturing practice (GMP) as set by KEBS, WHO, and Codex Alimentarius guidelines on dried fruit [27]. Baobab pulp from informal processors had significantly higher (*p* ≤ 0.001, *p* ≤ 0.001) Enterobacteriaceae and yeast and molds counts (3.1 ± 0.70 log_10_ CFU/g and 5.3 ± 0.11 log_10_ CFU/g) than those from formal processors (0.7 ± 0.29 log_10_ CFU/g and 3.1 ± 0.38 log_10_ CFU/g), respectively. Production practices, extrinsic, intrinsic, and processing factors determine the microbial load of food products at the time of consumption [28].

The total aerobic count loads in this study (4.3 ± 0.22) in pulp were higher than those of studies done on microbial analysis of freeze-dried fruits [18] and the commercial South African high-moisture dried fruits [17]. The TAC is primarily used to indicate bacterial populations, therefore informing on hygienic quality and compliance with good manufacturing procedures. Its presence in baobab pulp (4.3 ± 0.22) and candies (5.00 ± 0.22) implicates the baobab value chain's production processes. Microbiological quality may be compromised if processing is done in an unstandardized and unregulated manner. In addition, since there are no standard operating procedures during the cracking and scooping of the baobab fruits, preproduction contamination may be the greatest to the final poor microbiological quality [6].

The baobab pulp contamination can be traced back to handling practices along the value chain and at the farm level [14]. To meet the ever-rising baobab demand, farmers harvest fruits that have not attained the optimum drying index (Chadare et al. [7]). Most processors source baobab pulp from these farmers and traders who crack the fruits in the farms or aggregation centres. More often than not, these farmers and traders may not be conversant with the set hygienic guidelines. Hence, they crack the fruits in open and unhygienic undesignated areas around their homesteads and subject the pulp to open sun drying [14]. The open sun-drying process is a slow and time-consuming process that ends up exposing the pulp to insects, dust, rain, and other contaminants. Furthermore, slow drying results in insufficient drying of baobab fruits, and increased moisture content encourages the proliferation of fungal cells, which promotes the generation of mycotoxins (Bourdoux et al. [10]). The pulp contamination is exacerbated by the lack of proper packaging bags and unhygienic practices during handling, storage, and transportation to the final processors. A number of ready-to-eat products derived from baobab are not subjected to thermal processing, and hence, the chemical, physical, and microbial hazards throughout the value chain find their way into the end product. Hygienic handling and processing are therefore mandatory for the safety of baobab pulp [24].

A significant difference (*p* = 0.015) was observed in terms of Enterobacteriaceae counts between candies from formal (0.0 ± 0.00 log_10_ CFU/g) and informal processors (1.8 ± 0.56 log_10_ CFU/g). The Enterobacteriaceae counts in baobab products in this study were higher than those reported by Ntuli et al. [22], in Maseru, Lesotho. Enterobacteriaceae is frequently used as an indicator microorganism for the assessment of quality and hygiene conditions in the production and handling environment of food [29]. Its presence in high levels (3.1 ± 0.70 log _10_ CFU-g) (Table 1) in some of the baobab pulp from informal processors in this study could imply poor hygienic and sanitary conditions during processing [30]. Baobab candies had relatively low Enterobacteriaceae loads compared to the baobab pulp (Table 1). During processing, candies undergo thermal treatment where sugar, food coloring, and desired ingredients are dissolved in water and heated and then cooled and packaged (ICRAF [21]). Thermal treatment serves as a critical control point in the production of baobab candies where it significantly lowers the microbial load.

Although candies undergo a heat treatment that significantly lower the microorganism present in food, Enterobacteriaceae counts were still detected (1.80 ± 0.56 log _10_ CFU/g) in the candies from the informal processors (Table 1). Lack of sanitary hand washing facilities in production areas which are mostly around the homesteads may be the likely source of microbial contamination by the production personnel. During postheating stages such as cooling and packaging, microbes could be reintroduced to the finished products due to improper handling [28]. Poor practises along the baobab value chain such as poor storage conditions, poor packaging, unhygienic drying, and processing necessitate trainings and implementation of GAP and GMP along the value chain [22]. Although the informal sector has a large number of processors, their lack of knowledge might limit their ability to implement proper sanitary procedures along the baobab value chain. This is in contrast to the formal sector, where the Kenya Bureau of Standards, national training programs, and other organizations provide an opportunity for GHP and GMP training. Formation of processor groups might aid in accessing these trainings and possibly getting registered with KEBS.

Table 2 shows the mean comparison of the microbial content of baobab products sampled from Mombasa, Kilifi, and Nairobi. The analysis of variance showed significant differences (ANOVA, *F* = 5.51, *p* = 0.0068; *F* = 16.25, *p* = 0.0006; and *F* = 4.01, *p* = 0.036) in the total aerobic count, yeast and molds, and Enterobacteriaceae loads, respectively, among products from the regions. The highest TAC contamination in candies was detected in samples collected from Mombasa County (5.4 ± 0.28 log_10_ CFU/g), whereas the highest Enterobacteriaceae contamination was detected in samples collected from Kilifi county (3.3 ± 0.11 log_10_ CFU/g) (Table 2). This could be linked to the type of water used during processing [27]. Groundwater supplies 50% of the water demand in the counties of Kilifi and Mombasa [31]. Microbiological findings of groundwater in Kilifi County indicated the presence of *E. coli* in shallow wells and boreholes (Jimoh et al., [32]).

### 3.2. Fungal and Mycotoxin Contents of Baobab Products

The baobab pulp from informal processors in Mombasa and Kilifi recorded the highest yeast and mold contamination in baobab pulp at 5.4 ± 0.30 log_10_ CFU/g and 5.1 ± 0.01 log_10_ CFU/g, respectively. Similarly, candies from informal processors in Mombasa and Kilifi recorded the highest levels of yeast and molds at 4.3 ± 0.98 log_10_ CFU/g and 3.6 ± 0.05 log_10_ CFU/g, respectively, as shown in (Table 2). A significant difference was also observed in yeast and mold counts between pulp from informal processors in Nairobi (3.3 ± 0.52 log_10_ CFU/g) and those from Mombasa and Kilifi (5.4 ± 0.30 log_10_ CFU/g and 5.1 ± 0.01 log_10_ CFU/g), respectively. A similar trend can be seen in a study done on the effect of temperature on microbial growth in food during storage, where high environmental temperatures increase the proliferation of microorganisms [33]. Kilifi and Mombasa counties experience coastal temperatures which can be as high as 29 and 32°C, respectively. In addition, the mean annual humidity in both regions is 74 percent, with the most humid months reaching about 80 percent, which is conducive for mold growth. Furthermore, the plastic packages used for baobab candies and the majority of pulp from the informal sector may not be able to provide sufficient protection to prevent moisture-laden air from leaking in. In addition, these packages have very little resistance to internal pressure and can easily be pricked exposing the products to environmental contamination. This includes rendering the baobab candies and the pulp susceptible to moisture absorption which in turn favours the growth of yeast and molds. Safe moisture content and a relative humidity equilibrium have to be attained for long storage of foodstuffs to suppress the proliferation of fungi (Hell [34]).

Table 3 shows the ergosterol and aflatoxin content of baobab products while the correlations between levels of water activity, ergosterol, and aflatoxins are presented in (Table 4). All aflatoxin-contaminated baobab samples had concentrations that were higher than the maximum tolerable limits for the East Africa Community (EAC) standards of 5 *μ*g/kg for AFB1 and 10 *μ*g/kg for total aflatoxins. The EU standard for baobab pulp prescribes a maximum limit of 2 *μ*g/kg for AFB1 and 4 *μ*g/kg total aflatoxins. Temperature, water activity, and humidity play a vital role in fungal growth and mycotoxin production [35]. A positive correlation was observed between water activity and the ergosterol content among the baobab products (*r* = 0.5019, *p* = 0.04) (Table 4). There was also a positive correlation between the amount of ergosterol and amount of AFB2, AFG1, and AFG2 in baobab samples (*r* = 0.8362, *p* ≤ 0.001), (*r* = 0.8692, *p* ≤ 0.001), and (*r* = 0.8393, *p* ≤ 0.001), respectively. The results obtained are comparable to the prediction of mycotoxin deoxynivalenol (DON) contamination in Fusarium-infected wheat grains based on the determination of the ergosterol content [36]. The positive correlations observed between the water activity, ergosterol, and aflatoxin B2, G1, and G2 in contents in baobab samples (Table 4), respectively, shows that the quantitative measure of ergosterol is a good indicator of aflatoxin infection in food products [36].

Samples 1, 7, 12, and 14 had a water activity of above 0.7 (Table 3), which can easily provide a suitable environment for fungal growth as illustrated in the correlation (Table 4) [37]. Baobab traders in areas with elevated humidity and temperatures such as Kilifi and Mombasa can be disadvantaged if they are not able to control the water activity and moisture content. Samples 3, 5, and 16 had a water activity of less than 0.7 (Table 3); however, they displayed fungal and mycotoxin contamination, and this could be due to direct fungal contamination. Along the baobab value chain, some steps predispose baobab fruits to direct fungal contamination. Soil is the principal reservoir for fungal species inclusive of mycotoxin-producing fungi [35]. These fungi can invade baobab fruits during cracking and drying since these handling practices are done in the open in the farms or in collection centres. Besides, policies to ensure safe transportation of food products are not enforced, and therefore, baobab's pulp is transported in any available transportation truck that provides cheap services. This predisposes the baobab pulp to environmental fungal contamination [38].

**Figure 1:**
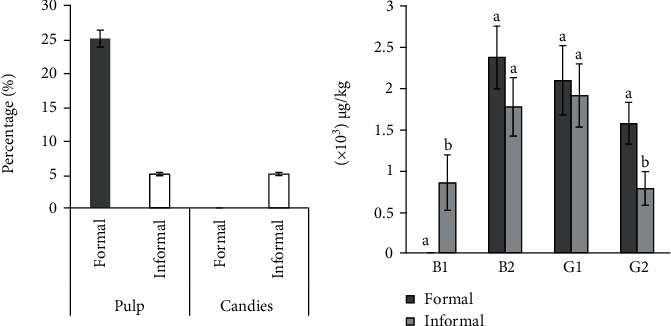
The percentage of aflatoxin-contaminated samples from formal and informal baobab processors and the mean comparison of the aflatoxin B1, B2, G1, and G2 between formal and informal processors products. Attached bars with same letters on top show no significance while bars with different letters on top show significant differences. The significance levels were considered at *p* ≤ 0.05.

The percentage of the fungal and aflatoxin-contaminated baobab samples is shown in Figure 1. There was no aflatoxin detection in baobab candies from formal processors. There was no significant difference in AFB2 and AFG1 contamination between baobab samples from formal and informal processors, whereas for AFB1 and AFG2, a significant difference was observed (Figure 1). The formal processors retail their products at 3000 KES (~$30) for a 200 grams og product, which could contribute to low turnover of products, prolonged shelf storage, and thus susceptibility to fungal growth and mycotoxin production. On the contrary, 200 grams of baobab pulp from informal processors retails at 50 KES (~$0.5). This leads to rapid turnover of these baobab products due to the affordability among the larger low-income consumer market segment.

The results from the study show that there is a minimal inspection of the on-shelf baobab products by the regulatory agencies. Aflatoxin concentrations were ranging between 3.93 and 11.09 × 103 *μ*g/kg in the analyzed samples indicating a potential health risk to baobab consumers [19]. The consumption of baobab candies and pulp is spread out among the young, the old, the pregnant, and the immune-compromised consumers. Therefore, no population group is excluded from the risks associated with the aflatoxin contamination in baobab products. Aflatoxin-contaminated baobab candies may have an impact on growth among children in the long run [28]. Another downside of having aflatoxin-contaminated pulp is that baobab products from the country of origin can be quarantined in the export markets with stricter mycotoxin regulatory controls. It is an exporter mandate to supply commodities with mycotoxins levels not exceeding the set maximum limits [28].

## 4. Conclusion

In conclusion, informal baobab processor products recorded the highest microbial contamination in terms of Enterobacteriaceae and TAC, which could be associated with unhygienic handling during processing. The results of this study incriminate the unhygienic postharvest practices along the baobab value chain as the source of contamination of the baobab pulp and candies. Therefore to ensure the safety of dried baobab fruits and baobab products along the baobab value chain, remedial steps such as training on good hygiene and good manufacturing practices as well as Hazard Analysis Critical Control Points (HACCP) should be taken [39].

### 4.1. Recommendation

To maintain the postharvest quality of baobab fruits, right postharvest management steps should be taken. For instance, baobab drying should be carried out using certain methods, such as convective air drying and vacuum drying, in an enclosed environment. However, if solar drying is to be used, the use of indirect solar drying or enclosed solar dryers is recommended as the product is covered with a transparent cover in a cabinet that protects it from dust, insects, rains, and rodents that could otherwise lead to contamination. Appropriate primary and secondary packaging technologies should also be incorporated especially hermetic bag packages.

## Figures and Tables

**Table 1:** Intrinsic properties and microbial content of baobab products from formal and informal processors.

Product	Source	Moisture content (%)	Range	*a*_W_	Range	Tac (log_10_ CFU/g)	*E* (log_10_ CFU/g)	YM (log_10_ CFU/g)
Baobab pulp	Formal	11.84 ± 2.30^a^	7.73-15.06	0.652 ± 0.07^a^	0.532-0.740	3.08 ± 0.08^a^	0.70 ± 0.29^b^	3.10 ± 0.38^b^
	Informal	13.45 ± 1.90^a^	10.50-5.60	0.695 ± 0.04^a^	0.585-0.741	4.30 ± 0.22^b^	3.10 ± 0.70^a^	5.30 ± 0.11^a^
*p* value		0.165		0.287		0.05	0.0008	0.0006
Baobab candies	Formal	11.28 ± 2.60^b^	8.99-13.57	0.619 ± 0.10^b^	0.551-0.687	5.00 ± 0.24^a^	0.00 ± 0.00^b^	3.50 ± 0.46^a^
	Informal	17.18 ± 3.80^a^	14.66-3.47	0.704 ± 0.06^a^	0.709-0.751	3.60 ± 0.27^a^	1.80 ± 0.56^a^	3.80 ± 0.25^a^
*p* value		0.014		0.05		0.65	0.015	0.49

Key: *a*_W_: water activity; SD: standard deviation; Tac: total aerobic count; *E*: *Enterobacteriaceae* counts; YM: yeast and molds counts. Values are means of two duplicate replicates, and those with the same superscript along the column for each baobab products are not significantly different at *p* ≤ 0.05.

**Table 2:** Comparison of the microbial content of baobab products based on region.

Product	Source	Region	log_10_ CFU/g Tac	log_10_ CFU/g *E*	log_10_ CFU/g Y+M
Baobab pulp	Formal	Nrb	4.4 ± 0.98^b^	0.5 ± 1.20^a^	3.6 ± 1.10^b^
		Msa	4.2 ± 0.04^b^	0.3 ± 0.60^a^	1.2 ± 0.05^a^
	Informal	Msa	5.4 ± 0.28^b^	2.9 ± 2.30^a^	5.4 ± 0.30^b^
		Kilifi	3.9 ± 0.21^a^	3.3 ± 0.11^b^	5.1 ± 0.01^b^
Baobab candies	Informal	Nrb	3.4 ± 0.51^b^	2.4 ± 0.08^a^	3.3 ± 0.52^a^
		Msa	2.4 ± 0.10^a^	1.4 ± 0.20^a^	3.6 ± 0.05^ab^
		Kilifi	4.2 ± 0.81^b^	2.1 ± 2.50^a^	4.3 ± 0.98^b^

Key: SD: standard deviation; Tac: total aerobic count; *E*: Enterobacteriaceae; Y+M: yeast and molds; Nrb: Nairobi; Msa: Mombasa. Values are means of two duplicate replicates, and those with the same superscript along the column for each baobab products are not significantly different by Bonferroni test (*p* ≤ 0.05).

**Table 3:** Ergosterol and aflatoxin content (mean ± SE) of baobab products.

Intrinsic properties			Ergosterol (mg/100 g)	Aflatoxin (×103 *μ*g/kg)			
Sample code	Moisture	*a*_W_		AF B1	AFB2	AF G1	AFG2
Pulp—formal							
S7	15.06 ± 0.09	0.74	1.68 ± 0.13	ND	7.69 ± 0.00	10.33 ± 0.03	5.13 ± 0.03
S5	11.49 ± 0.33	0.62	0.91 ± 0.08	ND	7.75 ± 0.00	8.32 ± 0.05	5.11 ± 0.03
S3	11.46 ± 0.52	0.61	1.06 ± 0.05	ND	7.68 ± 0.03	8.48 ± 0.14	5.09 ± 0.00
S1	14.98 ± 0.19	0.74	0.67 ± 0.04	ND	7.68 ± 0.03	ND	5.11 ± 0.03
Informal							
S16	12.30 ± 0.34	0.64	1.87 ± 0.04	ND	7.69 ± 0.00	8.23 ± 0.03	ND
Candies –informal							
S12	18.80 ± 0.16	0.749	1.92 ± 0.02	ND	5.91 ± 0.03	6.39 ± 0.05	3.93 ± 0.02
S14	14.65 ± 0.35	0.71	0.46 ± 0.19	11.09 ± 0.30	7.69 ± 0.00	8.34 ± 0.03	5.09 ± 0.00

Notes: AF: aflatoxin; ND: not detected; results are expressed as mean ± SD of three replications; aflatoxin contents are given in *μ*g/kg.

**Table 4:** Correlations between water activity, ergosterol, and aflatoxin content in baobab products.

	*a*_W_	Ergosterol	Aflatoxin			
			B1	B2	G1	G2
*a*_W_	1.000					
Ergosterol *p* value	0.5019 0.0476	1.0000	.			
B1	.	.	.			
B2 *p* value	0.4038 0.1209	0.8362 ≤0.001	.	1.0000		
G1 *p* value	0.1415 0.6012	0.8692 ≤0.001	.	0.7688 0.005	1.0000	
G2 *p* value	0.4077 0.1170	0.8393 ≤0.001	.	1.0000 ≤0.001	0.7703 0.005	1.0000

## Data Availability

The original research generated data used to support the findings of this study are included within the article.
